# Olive Pomace and Pâté Olive Cake as Suitable Ingredients for Food and Feed

**DOI:** 10.3390/microorganisms10020237

**Published:** 2022-01-21

**Authors:** Paola Foti, Alessandra Pino, Flora V. Romeo, Amanda Vaccalluzzo, Cinzia Caggia, Cinzia L. Randazzo

**Affiliations:** 1 Department of Agriculture, Food and Environment, University of Catania, Via S. Sofia 100, 95123 Catania, Italy; paola.foti@phd.unict.it (P.F.); alessandra.pino@unict.it (A.P.); amanda.vaccalluzzo@unict.it (A.V.); cranda@unict.it (C.L.R.); 2 ProBioEtna SRL, Spin off of University of Catania, Via S. Sofia 100, 95123 Catania, Italy; 3 Consiglio per la Ricerca in Agricoltura e l’analisi dell’Economia Agraria (CREA), Centro di Ricerca Olivicoltura, Frutticoltura e Agrumicoltura, Corso Savoia 190, 95024 Acireale, Italy; floravaleria.romeo@crea.gov.it

**Keywords:** olive oil extraction, DMF, by-products, bioactive compounds

## Abstract

Olive oil extraction generates several by-products that represent an environmental issue, mainly for Mediterranean countries where olive oil is mostly produced. These by-products represent an ecological issue for their phenolic components, such as oleuropein, hydroxytyrosol, and tyrosol. However, olive oil by-products can be treated and properly exploited in different fields for their health-promoting properties, and they represent great potential for the food and beverage, cosmetic, and pharmaceutical industries. Furthermore, recovery and treatment processes can contribute to efficient waste management, which can enhance the sustainability of the olive oil industry, and in turn, lead to relevant economic benefits. The solid waste, i.e., olive pomace, could be considered to be a suitable matrix or primary resource of molecules with high added value due to their high phenolic content. Olive pomace, at different moisture contents, is the main by-product obtained from two- or three-phase extraction systems. A commonly used centrifugal extraction system, i.e., a multiphase decanter (DMF), does not require the addition of water and can generate a new by-product called pâté or olive pomace cake, consisting of moist pulp that is rich in phenols, in particular, secoiridoids, without any trace of kernel. Although several reviews have been published on olive wastes, only a few reviews have specifically focused on the solid by-products. Therefore, the aim of the present review is to provide a comprehensive overview on the current valorization of the main solid olive oil by-products, in particular, olive pomace or pâté olive cake, highlighting their use in different fields, including human nutrition.

## 1. Introduction

The olive oil market is constantly growing and strongly subjected to technological innovation aimed to improve the yield and quality of the final product. The European countries are the main producers, consumers, and exporters of olive oil, providing about 67% of the world’s production. The cultivation of olive trees, combining traditional, intensive, and super intensive groves, in the EU Mediterranean countries, takes up about 4 million hectares. Among the EU, the largest consumers of olive oil are Italy and Spain, with an annual consumption of approximately 500,000 tons each, while Greece shows the highest per capita consumption, with about 12 kg per person per year [1].

As a result, there is an inevitable production of by-products coming from olive oil processing, i.e., mainly olive mill wastewater and olive pomace (OP). The valorization of these by-products responds to the strong demand for innovation in food system. Agro-food wastes and by-products represent a suitable matrix to be exploited in the food chain. This process involves the stabilization of wastes and by-products, and the extraction of added-value compounds that could be entirely treated or directly added as functional ingredients for designing new foods or valuable ingredients with medium- and high-added value [2]. Since the highest content (in the order of 70%) of phenolic compounds is found in the unwrapped part and in the outer parts of the olive fruit [3], several studies have focused on the detection of phenolic content in olive oil by-products [4,5].

Recently, nutrigenomic approaches have largely confirmed the beneficial effect of phenols, and highlighted their role in modulating the expression of different transcripts and mRNAs involved in glucose/lipid metabolism, proliferation, inflammation, and cancer [6]. The olive oil extraction process involves mainly pressure and centrifugation, and can be classified into two-phase and three-phase systems. Depending on the applied extraction process, different effluents are produced (Table 1). While the three-phase system requires high amounts of water and produces considerable amounts of effluent together with a pomace consisting of woody endocarp and cuticle with a moisture content of 48–54%, the two-phase system produces wet pomace with a moisture content of 58–62% [7]. Olive pomace (OP) has been mainly subjected to hexane extraction of residual oil and the obtained product then subjected to distillation and refining. However, the extraction of oil from OP has been discontinued because it is unprofitable and because it poses issues from a management point of view. For these reasons, several attempts have been made to use OP for the extraction of high added-value compounds or for direct use. To date, OP is used in agriculture as a soil conditioner and fertilizer; in bioenergy production; and for the extraction of hydroxytyrosol, tyrosol, oleuropein, caffeic acid, and squalene, intended for pharmaceutical, food, or cosmetic industries. However, up to now, no evidence of using such a product in human nutrition has been reported. Furthermore, an additional new by-product, called pâté olive cake (POC), is generated when olive oil extraction is carried out by using a multiphase decanter (DMF, Leopard, Pieralisi), which is an evolution of the two-phase system that combines the advantages of processing without water addition with the simplicity of three-phase extraction. The POC consists of olive pulp and vegetation water (with a moisture content of 75–90%) without any trace of kernel and it is the third product obtained by a DMF decanter together with virgin olive oil and a dried pomace (with a moisture content of 45–55%). POC is rich in bioactive compounds, and therefore, represents a promising matrix [8], potentially suitable for the formulation of new functional food. However, to reduce its bitter taste and its high perishability, due to high-water content, further treatments are required.

## 2. Physico-Chemical and Microbiological Characteristics of Olive Solid Waste

### 2.1. Physico-Chemical Traits of OP

OP is a solid waste that results from olive oil extraction and consists of pulp and kernel [10]. For one ton of processed olives, 0.5–0.6 tons of OP, characterized by high moisture content, are produced. The OP chemical composition is influenced by the olive cultivar, growing conditions, and by the extraction process used. Generally, the process of olive oil extraction produces, essentially, two types of OP, wet pomace (coming from a two-phase system) and dry pomace (coming from a three-phase system). The OP exhibits a dark color and pH values ranging between 4.8 and 5.2. The main components of fresh OP are water, carbohydrates, fiber, cellulose, hemicellulose, and lignin, although fats and proteins are also present, even if at smaller quantities [11]. Recently, Nunes et al. [12] reported the chemical composition of fresh OP, expressed as g/100 g. In particular, the moisture content was found to be around 60/100 g, carbohydrate content as 34/100 g, protein and ash as 2.6 and 0.7/100 g, respectively, and total fat content as 2.0/100 g. Regarding carbohydrates, they were mainly represented by lignin (43.95/100 g), insoluble and soluble fraction (26–17/100 g), hemicellulose (11.29/100 g), cellulose (9.55/100 g), and to a lesser extent, by arabinose, galactose, and mannose [13]. Furthermore, OP is characterized by a high content of both organic matter and carbon, high level of potassium, low content of phosphorus, and intermediate levels of nitrogen [14,15,16].

### 2.2. Microbiological Traits of OP

The microbiome of OP has been described as very similar to that of other oil by-products, such as OMWW, and composes both bacteria and yeasts. Vivas et al. [17] identified *Proteobacteria* as a dominant member, followed by *Actinobacteria (Streptomyces), Firmicutes (Staphylococcus)*, and *Acidobacteria*. In addition, members of *Hydrocarboniphaga, Pseudoxanthomonas*, and *Stenotrophomonas (Gammaproteobacteria)* were detected, with *Comamonas (Betaproteobacteria)* as the main microbial group. In addition, the bacterial population has been explored by amplification of the internal transcribed spacers between the 16S and 23S rRNA genes (ITS-PCR) and by 16S rRNA sequencing. The results showed that *Firmicutes* were the most prevalent and diverse members [18]. Regarding the fungal population, it seemed to be strongly influenced by the olive cultivar. The dominant yeasts were *Pichia caribbica* (syn. *Meyerozyma caribbica*), *Pichia holstii* (syn. *Nakazawaea holstii*), and *Zygosaccharomyces fermented* (syn. *Lachancea fermenta*), followed, to a lesser extent, by *Zygosaccharomyces florentinus* (syn. *Zygotorulaspora florentina*), *Lachancea thermotolerans* (syn. *Kluyveromyces thermotolerans*), *Saccharomyces cerevisiae*, and *Saccharomyces rosinii* (syn. *Kazachstania rosinii*).

### 2.3. Physico-Chemical Traits of POC

DMF (multiphase decanter, Leopard) extraction technology is the only two-phase centrifugal system that produces a dehydrated pomace and recovers the POC inside the drum. This by-product shows a semi-solid consistency, and its composition is strongly influenced by the olive cultivar and ripening period. It is essentially composed of olive pulp, olive skin, wastewater, and devoid of woody parts [19]. In detail, the POC presents a high content of organic matter, fiber, and crude protein, and a low lignin content, similar to OP obtained from pitted olives. Recently, Lanza et al. [9] reported the composition of POC obtained from different cultivars such as Leccino, Carboncella, and Tortiglione. The titratable acidity, expressed as g/100 g of citric acid, ranged from 0.29 to 0.56, and the pH value ranged from 4.95 to 5.20. The moisture percentages were found to be between 81 and 84%, and the ash content was from 1.57 to 2.75%. The residual oil content, determined by Soxlet, ranged from 3.7 to 4.2%. A higher residual oil content of 10% and a lower moisture content (as 77%) was found in Frantoio and Leccino cultivars. Furthermore, the POC was characterized by high levels of essential fatty acids, such as linoleic and linolenic acids (8.5% and 1%, respectively) [8].

### 2.4. Microbiological Traits of POC

Currently, few studies have been carried out on microbiological characterization of POC. As recently reported by [9], the results of microbiological analyses, carried out on MacConkey agar for *Enterobacteriaceae* counting, on malt extract agar for yeasts and molds, and on Man, De Rogosa, and Sharpe agar for lactic acid bacteria (LAB) counting, showed cell density values, expressed as colony forming units (CFUs), of around 10^5^, 10^4^, and between 10^3^ and 10^4^ CFU/g, respectively.

## 3. Bioactive Compounds of Solid Oil Waste Products

The use of vegetable by-products for the recovery of bioactive substances through chemical and biotechnological processes is a promising strategy [20]. The bioactive molecules found in food waste are known for their antioxidant and radical scavenging activity; for inhibiting the oxidation of DNA, proteins and lipids; and for significantly reducing the development of several diseases, such as cancer, Alzheimer’s, and Parkinson’s [21]. Furthermore, the OP is widely used in different fields for antioxidant and antimicrobial properties [22]. Several phenolic compounds have been detected by liquid chromatography techniques coupled with quadrupole time-of-flight mass spectrometry (QqTOF/MS) in OP such as: oleuropein, hydroxytyrosol and tyrosol derivatives, iridoid precursors, secoiridoids and derivates, flavonoids, lignans, and phenolic acids. In particular, the most abundant phenolic compound present in olive fruit, namely the oleuropein, has been found at high concentrations in OP, reaching concentrations up to 0.9% [23,24]. The main antioxidants identified in the OP were vitamin E (α-, β-, γ-tocopherol, and α-tocotrienol); among them, α-tocopherol was the major vitamin E form (2.6 mg/100 g of raw matter). Concerning the POC obtained from the DMF system, different compositions have been reported in the literature, probably due to the different olive cultivars used for oil extraction. According to some authors, unlike other olive oil by-products, POC is mainly composed of oxidized hydroxytyrosol and luteolin, while other authors have reported mainly oleacein, hydroxytyrosol, and verbascoside. However, significant differences have been found among the different cultivars: while the POC obtained from Carboncella and Tortiglione cultivars showed the highest values of hydroxytyrosol, i.e., 977 and 720 mg/kg, respectively, and tyrosol, i.e., 57 and 67 mg/kg, respectively, the Leccino cultivar showed lower values, i.e., 243 and 21 mg/kg [9]. Furthermore, Cecchi et al., 2018, confirmed that the main components of POC are hydroxytyrosol, tyrosol, 3,4- DHPEA-EDA, and verbascoside.

## 4. Exploitation of OP

OP has been traditionally used as a matrix to recover residual oil by solvent extraction (usually hexane) and the obtained spent pomace has been intended as a fuel or for use in the cabinetmaking sector [22]. Oil extraction from pomace is an expensive method and the use of this by-product has been proposed in different fields, in particular, in agriculture, in bioenergy production, and as a matrix for extraction of high added-value components to be used in the food, feed, and packaging sector. Regarding their use in agriculture, Innangi et al. [25] showed that despite the content of phenolics and salts, direct dispersion of OP in soil did not entail any negative impact. As a matter of fact, the long-term use of OP seemed to improve organic matter, enzymatic activities, and the soil quality index. Furthermore, several authors demonstrated that direct application to soil could entail an increase in minerals, such as nitrogen, useful for crop production and could remove pollutants, such as heavy metals, herbicides, and triazine herbicides [26]. According to the Italian law [27], the spreading of OP must follow a suitable distribution and absorption and the recommended dosage is of 10 m^3^/ha. As far as the production of bioenergy is concerned, the combustion of OP results in lowering the costs of energy conversion from non-renewable sources, for generation of thermal and electrical energy [28]. Several studies have demonstrated that aerobic digestion of OP showed good energy recovery. Furthermore, anaerobic digestion of biomass could produce biogas and recover energy. In addition, the indigenous microorganisms of OP have been evaluated for ethanol production and a 3% yield has been reported [29].

## 5. Use of Solid Olive By-Products in the Food and Feed Sectors

### 5.1. Use of OP in Food

Several applications of OP in the food and feed sectors have been proposed. In the food sector, OP has been added as an extract to preserve and increase final product stability. OP powders represent a matrix rich in various nutrients and bioactive compounds with human health and technological effects, as rheological improvement of final products [30]. However, despite their antioxidant and antimicrobial activity, the addition of vegetable by-products could alter the sensorial and technological properties of food, and for this reason, the specific amount to be added in formulations must be carefully selected [31]. An effective strategy to avoid the sensorial alteration of final products is the encapsulation of bioactive molecules. Such a strategy, in addition to improving their functional properties, provides adequate protection from thermal insults (temperature increasing), controls their release into the food matrix, and reduces the amount needed to be effective in food applications [32]. Phenols extracted from OP have been added to olive oil to further improve healthy trait and to inhibit lipid oxidation. Although results have shown increased oxidative stability of the final product, an alteration of sensory traits was observed, with bitterness and off-flavor significantly higher in phenol-enriched virgin olive oil. Regarding bakery products, OP is added (at 10% *w*/*w*) in bread and pasta. Cedola et al. [33] demonstrated that although the addition of both olive wastewater and OP improved the nutraceutical value of final product, the OP resulted the most suitable ingredient despite higher was the adverse effect on sensory traits, related to bitter and spicy taste. Simonato et al. [34] fortified pasta by replacing durum wheat semolina with different concentration of OP (0.5 and 10 g/100 g). Fortification with OP significantly increased the content of total phenolics, and consequently, the antioxidant activity both before and after cooking. Furthermore, the OP fiber content resulted in an in vitro change in the digestibility of starch, by decreasing the rapidly digestible fraction and by increasing the slowly digestible starch fraction. Moreover, OP flour has been added to biscuits at 15% to develop functional foods with higher fiber content, high nutritional value, fewer calories, and lowered glycaemic index [35]. In a further study, volunteers eating OP-supplemented biscuits exhibited a shift in their intestinal microbiota. A metagenomic analysis of 16S rRNA profile showed an increase in the abundance of Akkermansia and Bifidobacterium, both positively correlated to host physiology and protection from metabolism and cardiovascular disease [36,37]. In addition, Bacteroides and Prevotella, linked to the onset of obesity and traditional dietary paradigms, were also positively affected, whereas Enterobacteriaceae were negatively correlated [36,37,38]. In addition, an LC-MS analysis detected significant increases in phenolic acid concentrations in urine, 24 h after ingestion of enriched OP biscuits. Homovanillic acid and hydroxytyrosol derivates, involved in oxidative LDL-cholesterol reduction, were detected in blood [36]. These findings were in agreement with those reported by Ribeiro et al. [39], who confirmed the prebiotic activity of OP-added formulations subjected to simulated gastrointestinal digestion followed by in vitro fecal fermentation. In particular, the formulation obtained from the liquid fraction induced a positive effect on the Prevotella spp./Bacteroides spp. ratio, resulting in production of short-chain fatty acids and showing good mucin-adhesion inhibition capacity against Bacillus cereus (22.03 ± 2.45%) and Listeria monocytogenes (20.01 ± 1.93%). Other studies have reported that the addition of polyphenol-rich extracts to dairy products increased their stability and prevented rancidity [40]. The phenolic extract was added to fermented milk at 100–200 mg/L ratio. The results showed that this addition did not interfere with the fermentation by Streptococcus thermophilus TA040 and Lactobacillus acidophilus LAC4 strains. Different concentrations (2, 4, 6, and 8 mg/100 g) were also added to butter and two storage temperatures (25 and 60 °C) were evaluated. The results showed that the highest tested concentration conferred resistance against oxidative stress [41]. Furthermore, Ribeiro et al. [42] demonstrated that the addition of OP extracts (1–2%) in yoghurt provided 5 mg of hydroxytyrosol per day, increased fiber content, and improved the bioaccessibility of total phenols by 25.58%.

### 5.2. Use of OP in Feed

The use of OP for feed purposes is a widespread practice for the re-use of this by-product. In order to have a good market value, a feed must provide both adequate nutritional value and low cost, in order to compete with conventional feeds [43]. The addition of olive by-products in feed, used in both aquaculture and livestock, did not show any adverse effect on animal growth and improved the fatty acid profile by reducing saturated acids [44].

In aquaculture feed, the partial substitution of any of the most common vegetable oils (such as soybean, linseed, sunflower, rapeseed palm oil, and olive oil) can be proposed only if fatty acids in diet are adequately present to meet the essential fat requirements of fish and ultimately humans. Sioriki et al. [45] demonstrated that OP added at 8% in sea bream feed improved the cardioprotective properties of the final product by enriching the lipid profile of the fish with specific cardioprotective lipid compounds of vegetable origin. In addition, other authors have integrated OP in the diets of sea bass and sea bream and showed a presumed cardioprotective effect evidenced by an increased production of eicosapentaenoic acid (EPA) and docosahexaenoic acid (DHA) in the lipid fraction of fish [46,47]. OP has been added to rabbit and lamb feed. Specifically, the introduction of OP into lamb feed increased the oxidative stability of the meat [48]. Similar results were observed for lambs [49]. The addition of 35% of OP and linseed to animal feed has shown a synergistic effect by increasing the level of polyunsaturated fatty acids and vitamin E, and decreasing the level of peroxides, thiobarbituric acid reactive substances (TBARS), and the number of conjugated dienes. In addition, oxidative stability has been monitored in pork. Phenols were detected in the meat, where they increased the sensory characteristics of the product and a 5% decrease in saturated fatty acids was observed [50,51]. Furthermore, several studies have shown that the addition of OP to animal feed is beneficial to animals and also improves the nutritional quality and quantity of the by-products, i.e., dog, milk, cheese, and eggs. Chiofalo et al. [52] demonstrated that the addition of OP, between 7.5% and 15%, positively influenced the performance of animals, increasing meat tenderness and influencing meat quality indices, such as intramuscular fat and unsaturated fatty acids. In addition, milk produced from cows fed with fortified feed showed an increase in unsaturated fatty acids content (oleic acid, vaccenic acid, and CLA) and a decrease in SFA (short- and medium-chain fatty acids). These findings suggest that OP can improve the nutritional properties of milk and cheese without compromising their sensory profile [52]. Results related to fatty acid composition of cow’s milk and cheese were confirmed by Castellani et al. [53], who indicated that monounsaturated and polyunsaturated fatty acids were increased by supplementing the feed with OP. In order to assess any shift in the flavor profile of milk or dairy products obtained from cows fed with OP, analyses were carried out using GC/MS and electronic nose. The results showed that OP did not alter the sensory profile of the product. Several studies have shown that milk produced by sheep and buffalo induced nutritional improvements in the product itself. In fact, Mannelli et al. [54] and Vargas-Bello-Pérez et al. [55] demonstrated that the addition of OP in feed improved the nutritional characteristics of sheep milk. In particular, the revealed microbiota composition showed a reduction in Anaerovibrio, a lipase-producing bacterium, which induced an inhibition of lipolysis at the expense of polyunsaturated fatty acids. Chiofalo et al. [56] also evaluated the yield and composition of ewe’s milk and highlighted that the OP addition positively influenced the milk yield and improved the nutritional values by increasing the unsaturated to saturated fatty acids ratio. Finally, the use of OP at 10% also seems to have a positive effect on laying hens. Indeed, the results of a transcriptomics analysis showed that the cholesterol content of eggs was decreased as compared with controls, probably due to the modulatory effects of phenols on genes involved in the cholesterol biosynthesis pathways [57].

### 5.3. Use of DMF POC for New Functional Food

POC obtained from a two-phase extraction system consists of a moist pulp containing lipophilic and hydrophilic fractions, and is considered to be a matrix with functional and technological properties (Table 2). Currently, the main applications of POC are in the feed sector, although several studies have evaluated strategies to process this by-product into a product intended for human consumption, as a food or nutraceutical supplement, which could represent a profitable advantage for olive oil companies. As matter of fact, POC has been proposed as a valuable functional ingredient to fortify feed. Indeed, the inclusion of fresh POC (35%) in lamb feed demonstrated an improvement in meat quality without compromising oxidative stability [49]. Similar results were observed in chickens raised with PCO fortified feed. The results showed that the growth of chickens was better with the highest dose of POC (165 g/kg). Tyrosol and sulphate metabolites of hydroxytyrosol were found in the poultry meat and, consequently, an increase in the oxidative stability of the meat was observed [50].

POC has also been proposed as a functional additive for the formulation of bakery products. Padalino et al. [19] fortified spaghetti with POC flour at a rate of 10 to 15% *w*/*w*. The spaghetti fortified with 10% *w*/*w* showed a high added-value content of phenolic compounds and were judged acceptable from a sensory point of view. A similar study [58], carried out on taralli, a typical Apulian product, was performed using POC, previously subjected to sequential fermentation, with yeast and LAB (namely *Saccharomyces cerevisiae* and *Leuconostoc mesenteroides*). The taralli was enriched by adding 20% fermented POC from black olives. The profiles of both the bioactive compounds and the fatty acids were monitored during storage for 180 days. The results showed significantly higher levels of bioactive compounds (hydroxytyrosol, tyrosol, verbascoside, oleacin, oleocanthal, maslinic acid, *α*-tocopherol, and lutein) than the control. In addition, the enriched taralli maintained a low content of saturated fatty acids and a high level of polyphenols, for up to 90 days of storage. However, the use of POC for human consumption seems to be an increasingly interesting objective for the scientific community. Indeed, several authors have already proposed POC as a new food or nutraceutical supplement, demonstrating its beneficial effects on human health. In detail, as suggested by Cecchi et al. [59], based on the total phenolic content, 1 g of dried POC provided a daily intake comparable to 200 g of virgin olive oil [59]. Tuffariello et al. [60] proposed a new product coming from a sequential fermentation, on a pilot scale, through inoculation of S. cerevisiae and Leuc. mesenteroides. The sequential inoculum improved fermentation performance significantly and demonstrated that, as in the case of table olives, the first part of fermentation was dominated by yeasts and the second part by LAB. In fermented POC, the total phenol levels were slightly reduced as compared with an unfermented sample; however, the hydroxytyrosol content was higher, while triterpene acids, carotenoids, and tocochromanols remained unchanged. A desirable shift in volatile compounds, due to production of alcohols, esters, and acids during fermentation, was observed. The olive cultivar seems to be the most relevant factor that affects phenolic content and biological characteristics. Peršurić et al. [61] evaluated the biological activity of two POCs obtained from different cultivars, namely Frantoio and Ascolana tenera. The results obtained through chromatographic analysis (LC coupled to triple quadrupole mass spectrometry) and MALDI-TOF/MS showed that the POC presented a high content of hydroxytyrosol, verbascoside, and oleuropein aglycone derivatives and a content of triacylglycerols, rich in oleic fatty acid. The Frantoio variety, as compared with Ascolana, showed higher antioxidant activity, with phenol content of 26.66 and 17.48 g GAE/kg, respectively. The biological activity was evaluated on different enzymes, namely amylase and glucosidase, known as targets in diabetes mellitus and cholinesterase, and are the enzymes mainly involved in Alzheimer’s disease [62]. Overall, POC from Ascolana showed a higher tyrosinase and amylase inhibition as compared with Frantoio. Most recently, Lanza et al. [9] confirmed a significant correlation between phenolic content and the cultivar. In agreement with previous reports, the olive cultivar from which the POC was obtained, strongly influenced the bioactive compounds detected. In detail, POC obtained from Carboncella and Tortiglione cultivars showed a higher content of total biophenols (5899 and 5543 mg/Kg, respectively) as compared with that obtained from the Leccino cultivar which exhibited a concentration of 948 mg/Kg. In the same study [9], POC extract left to spontaneous fermentation was tested on CaCo2 and HCT116 colon cancer cells, through an MTS assay, which is a colorimetric method applied for the sensitive quantification of viable cells. The rate of inhibition of cell proliferation was affected by the different tested extracts and by the used dilutions, and these results were correlated to the different contents of each phenolic compound, such as oleuropein, verbascoside, hydroxytyrosol, and the secoiridoid derivative oleacein. Indeed, in Caco2 cells, POC obtained from the Leccino cultivar induced significantly higher cell viability as compared with control cells, at any tested concentration. A similar effect was observed on HCT116 cells with POC obtained from Carboncella and Tortiglione cultivars at specific concentrations. Furthermore, other studies have focused on assessing the antiaging effect and the cardioprotective activity of different POCs. In detail, Cecchi et al. [59] evaluated the phenolic content present in fresh and freeze-dried POCs. In fresh POC stored in tanks for 4 months, an increase in hydroxytyrosol content was observed over time, with concentrations reaching 5635 mg/Kg, whilst a decrease in oleuropein and verbascoside content was observed. In contrast, the freeze-dried POC was found to maintain the same phenolic content for several months. Tests with a hydroalcoholic extract from POC showed antiaging activity in human cells similar to the effect obtained by using pure hydroxytyrosol. Furthermore, the cardiovascular and metabolic protective effects of tablets obtained from POC (corresponding to 30 mg/day of hydroxytyrosol) was in vivo tested [63]. The results showed, in plasma, a reduction in total cholesterol (−10.8 mg/dL), LDL-cholesterol (−10.8 mg/dL), and urea, and a significant increase in calcium (+0.3 mg/dL). In addition, leukocytes, subjected to exogenous oxidative stress induced with H_2_O_2_, reacted by increasing the levels of the antioxidant transcription factor Nrf-2 by 88.9% and by reducing plasma levels of the proinflammatory cytokine MCP-1, a proinflammatory protein involved in the atherosclerotic process [63]. To explore the interaction between POC and human intestinal microbiota, a study using the SHIME^®^, an advanced gastrointestinal simulator, was proposed [64]. The study aimed to understand how the phenolic fraction present in POC influenced bacterial growth and how it could exert an antimicrobial effect in a dose-dependent manner. The main compounds found were: oleuropein-derived molecules, free hydroxytyrosol, and in a smaller amount, verbascoside and luteolin; while the fiber content was composed by both insoluble and soluble fraction (20.4% and 3.7%, respectively); whereas monosaccharide and protein contents were present at 16.8% and 9%, respectively. The same study [64] confirmed that the POC did not exhibit any antimicrobial effect on the intestinal microbial community, as the SCFA production was not reduced, while it induced a reduction in Fusobacteriaceae (usually related to inflammatory status) and an evident increase in Lactobacillaceae and Bifidobacteriaceae [64]. With regard to phenolic content, a decrease in hydroxytyrosol combined with a contextual increase in tyrosol, registered after 9 days, confirmed the presence of esterases, which are commonly active in gut microbiota and known to be involved in hydrolysis of various phenolic compounds.

**Table 2:** Different applications of POC.

Sector	Aim	POC-Form	Amount	Results	References
Livestock	Feed fortification	Fresh	35%	Reduction in oxidation of cholesterol and fatty acids in lamb	[49]
	Feed fortification	Fresh	82.5/165.0 g/Kg	Enhancement of meat oxidative stability at higher POC concentrations	[50]
Natural additives for food application	Increase the nutritional value of spaghetti	POC endured air-dried at low temperature	10 and 15% (*w*/*w*)	Increase in the content of flavonoids and total phenols	[19]
	Increase the quality and nutritional value of taralli	POC fermented with *S. cerevisiae* and *Leuc. mesenteroides*	20%	Increase in the bioactive compounds and saturated fatty acids maintained at a low level	[58]
Food	New fermented product	Fermented POC with *S. cerevisiae* and *Leuc. mesenteroides*	-	Increase in hydroxytyrosol content and improved sensory notes by production of alcohols and esters	[59]
	Formulation of new functional food	Fresh, fermented, or extracted POC		Carboncella, Tortiglione and Leccino cv. show different total phenol contents The metabolic activity, tested on CaCo2 and HCT116, suggest beneficial effects related to modulation of gene expression	[9]
Nutraceutical	Formulation of new functional ingredients	Fresh	-	The Frantoio cv show high antioxidant activity with a phenol content of 26.66 gGAE/kg The POC obtained from Ascolana cv show higher tyrosinase and amylase inhibition than those obtained from Frantoio cv.	[61]
Pharmaceutical	Evaluation of antiaging effect of phenolic extract	Fresh and dried	-	Dried POC contains high levels of hydroxytyrosol, oleuropein derivatives. and exhibited a long stability, unlike fresh POC, where a breakdown of hydroxytyrosol occurred The diluted hydroalcoholic extract shows an in vitro antiaging effect	[59]
	Evaluation of effect on cardiovascular and metabolic diseases	Tablet	4 tablets/day of POC (corresponding to 30 mg/day of hydroxytyrosol for 2 months)	Reduction in total cholesterol, LDL-cholesterol, and urea, and significant increase in plasma calcium levels Decrease in the proinflammatory protein MCP-1 in plasma	[63]
	Evaluation of interaction between POC and intestinal microbiota through SHIME^®^	Powder	4 g/L of POC	No antimicrobial effect on the colonic microbial community, as SCFA production is not reduced Reduction in *Fusobacteriaceae* and increase in *Lactobacillaceae* and *Bifidobacteriaceae*.	[64]

## 6. Conclusions and Future Perspectives

The valorization of agro-food waste is a challenging opportunity for the sustainable and competitive development of an innovative food system and the use of by-products in the food sector remains to be a relevant challenge, with a view to the creation of virtuose recycling.

Although a large number of studies have been published on olive oil by-product treatment and/or valorization, only a few studies have specifically focused on valorization of OP or POC in the food and feed sectors, highlighting that these solid by-products represent a valuable source of bioactive compounds which deserve to be reused. As a matter of fact, OP and POC applied as such or in low percentages in food formulations have proven to extend shelf-life, therefore, enhancing the functional traits of final products. Nowadays, the recovery of bioactive compounds and their addition to food formulations is a promising strategy to obtain functional food. The next challenge is the valorization of POC through biological debittering and stabilization to promote its rational use and to enrich the lipophilic and hydrophilic bioactive compounds in food, which can exert beneficial effects on human health.

## Figures and Tables

**Table 1:** Olive oil and by-products from different extraction systems [7,9].

Extraction System	Olive Oil (kg/100 kg olive)	Added Water (%)	Olive Mill Wastewater	Olive Pomace (kg/100 kg olive)	Pomace Moisture (%)
Traditional		-	40	40	20
Three phases	20	50	80–110	55–57	48–54
Two phases	20	0–10	8–10	75–80	58–62
Two phases-DMF	20	-	-	45–55	75–90
Three phases to savings	20	10–20	33–35	56–60	50–52
